# The Risk of Neuraxial Anesthesia-Related Hypotension in COVID-19 Parturients Undergoing Cesarean Delivery: A Multicenter, Retrospective, Propensity Score Matched Cohort Study

**DOI:** 10.3389/fmed.2021.713733

**Published:** 2021-08-19

**Authors:** Yuan Zhang, Rong Chen, Chen Cao, Yuan Gong, Qin Zhou, Min Wei, ZhongYuan Xia, XiangDong Chen, QingTao Meng

**Affiliations:** ^1^Department of Anaesthesiology, Renmin Hospital of Wuhan University, Wuhan, China; ^2^Department of Anaesthesiology, East Hospital, Renmin Hospital of Wuhan University, Wuhan, China; ^3^Department of Medical Center, Renmin Hospital of Wuhan University, Wuhan, China; ^4^Department of Anaesthesiology, Yichang Central People's Hospital, The First College of Clinical Medical Science, China Three Gorges University, Yichang, China; ^5^Department of Obstetrics, East Hospital, Renmin Hospital of Wuhan University, Wuhan, China; ^6^Department of Anaesthesiology, Union Hospital, Tongji Medical College, Huazhong University of Science and Technology, Wuhan, China

**Keywords:** neuraxial anesthesia, hypotension, COVID-19, cesarean delivery, propensity score matching

## Abstract

**Background:** SARS-CoV-2 infection was referred to sympathetic hyperactivity, which might increase the susceptibility of neuraxial anesthesia-related hypotension resulted from sympathetic inhibition. We conducted a multicenter, retrospective, propensity score matched (PSM) cohort study to determine whether COVID-19 parturients have an increased risk of hypotension after neuraxial anesthesia for cesarean delivery.

**Methods:** Clinical data of COVID-19 parturients were collected from the electronic medical records from 1th January to 31th May, 2020 in three hospitals of Hubei Province, China. Information of Control parturients (without COVID-19) were obtained at the same institutions over a similar period in 2019. All American Society of Anaesthesiologists (ASA) Physical Status II full termed pregnant women who received cesarean delivery under neuraxial anesthesia were included. The primary objective was to obtain and compare the incidence of neuraxial anesthesia-related hypotension. Secondary objectives were the analysis of anesthetic implementation and administration, intraoperative maternal vital signs and adverse reactions, and neonatal Apgar scores at 1 and 5 min after delivery. The clinical characteristics of COVID-19 parturients were also analyzed. PSM was derived to balance the predictors for neuraxial anesthesia-related hypotension based on previous studies.

**Results:** In present study, 101 COVID-19 parturients and 186 Control parturients were derived from 1,403 cases referenced to propensity score matching. The incidence of neuraxial anesthesia-related hypotension was 57.4% in COVID-19 parturients and 41.9% in Control parturients with an incidence risk ratio (IRR) of 1.37 (95% CI 1.08–1.74; *P* = 0.012; *post-hoc Cramér's V* = 0.15) in the PSM cohort. The incidences of nausea, vomiting, dizziness, and shaking were significantly higher in the COVID-19 group than Control group (48.5 vs. 17.2%, *P* < 0.001; 10.9 vs. 4.3%, *P* = 0.03; 18.8 vs. 3.2%, *P* < 0.001; 51.5 vs. 18.3%, *P* < 0.001; respectively). The Apgar scores at 1 min was significantly lower in newborns from COVID-19 parturients than that in Control babies (*P* = 0.04).

**Conclusions:** An increased risk of neuraxial anesthesia-related hypotension in COVID-19 parturients undergoing cesarean delivery should be stressed.

## Introduction

A novel coronavirus (SARS-CoV-2) infection disease (COVID-19) has devastated the global community since the end of 2019 (1). More importantly, mutations in SARS-CoV-2 might (partly) escape the immune response which led to a large drop in efficacy of vaccine. Several studies have forecasted a second rebound of COVID-19 would be manifested in countries with outbreaks (2, 3). We have to pay more attention to the current COVID-19 pandemic to cope with the unexpected medical scenarios.

Although the clinical characteristics of COVID-19 in pregnant women are similar with non-pregnant women (4), COVID-19 results in additional challenges for obstetric anesthesia as reported in previous study (5, 6). The most preferred method for cesarean delivery is neuraxial anesthesia [epidural anesthesia (EA), spinal anesthesia (SA), and combined epidural–spinal anesthesia (CES)] which allows parturients to remain respiration and avoids the effects of general anesthetic on newborns. Meanwhile, sympathetic blockade induced by neuraxial anesthesia can provoke maternal hypotension which has a potential risk for transient tachypnea of newborns (7). Several studies have examined the incidence and associated factors of hypotension after neuraxial anesthesia in healthy parturients undergoing cesarean delivery (8–11). However, parturients with COVID-19 have not been included in those studies.

Angiotensin-converting enzyme 2 (ACE2) has recently been identified as the SARS-CoV-2 receptor. Binding by SARS-CoV-2 attenuated the cardiovascular protection of ACE2 system, which closely linked with sympathetic overactivation and renin-angiotensin system overflow (12, 13). Besides, both psychological stress after being diagnosed with COVID-19 and emotional components associated with clinical isolation may further hasten sympathetic excitation (13). Enhanced sympathetic activity combined with hypoxemia induced by pulmonary inflammation would add significant stress to cardiovascular system. A latest study found the incidence of autonomic dysfunction (sympathetic, parasympathetic, or both) was 78.0% in mild COVID-19 patients, which resulted in a higher risk of orthostatic hypotension when compared with un-COVID-19 patients (14). Cardiovascular system complications in COVID-19 patients increasingly become a concern (15). However, the effects of SARS-CoV-2 infection on hemodynamics of parturients who underwent neuraxial anesthesia for cesarean delivery are still unclear.

In the present study, we conducted a retrospective analysis to obtain and compare the prevalence of neuraxial anesthesia-related hypotension during cesarean delivery in COVID-19 parturients and control parturients (without COVID-19).

## Methods

Ethical approval for this study (No. WDRY2020-K077) was provided by the Institutional Review Board at Renmin Hospital of Wuhan University, Wuhan, China (Chairperson Prof. Hong Chen) on 29 February 2020. The study received exemption from informed consent. All personal information were removed from the database to protect patients' confidentiality.

A multicenter, retrospective, propensity score matched cohort study was designed to compare the incidence of neuraxial anesthesia-related hypotension in parturients with and without COVID-19 undergoing cesarean delivery. The methodology reported in this study was accordance with the recommendations of the Strengthening the Reporting of Observational studies in Epidemiology (STROBE) statement (16).

Renmin Hospital of Wuhan University, Union Hospital Affiliated to Tongji Medical College of Huazhong University of Science and Technology, and Yichang Central People's Hospital were designated as the diagnosis and treatment center for COVID-19 patients (including pregnant women) in Hubei province during the pandemic. SARS-CoV-2 nucleic acid test was used to screen COVID-19 in all parturients. And the chest CT scan was performed on parturients after delivery. We planned to collect all of the available COVID-19 parturients undergoing cesarean delivery who met the inclusion/exclusion criteria in three hospitals from 1th January to 31th May, 2020. And, information of control parturients (without COVID-19) were obtained at the same institutions over a similar period in 2019. We identified patients with a primary International Classification of Diseases, Tenth Revision (ICD-10) diagnosis code of cesarean delivery from the electronic medical records to establish the study cohort. All ASA Physical Status II full termed pregnant women who received cesarean delivery (ICD-10 codes O82.0–O82.9, and O84.2 for cesarean delivery) under neuraxial anesthesia were eligible for inclusion in the study. Parturients who had a cesarean delivery after failed vaginal delivery were also included. The exclusion criteria included severe complicated births, significant bleeding (intraoperative bleeding over 1,000 ml), inadequate blockade (requiring addition of a general anesthetic administration) or incomplete data. The same inclusion and exclusion criteria were used for both two groups in present study.

A *post-hoc* estimated effect size (*Cramér's V*) for the study was assessed by comparing the incidence of neuraxial anesthesia-related hypotension between parturients with and without COVID-19 before and after propensity score matching (PSM). The clinical data including patient demographics, anesthesia management, and intraoperative records were independently collected using prefabricated forms and cross checked by two investigators in each institution in order to maintain the quality and consistency of data.

Neuraxial anesthesia protocols for cesarean delivery are similar in three institutions. Before initiation of anesthesia, an intravenous line, ECG, pulse oximetry, and non-invasive automatic blood pressure monitors (1- or 2-min interval) were placed. The puncture procedure was performed in the left lateral decubitus position. Block height was assessed bilaterally using loss of cold sensitivity to alcohol at an interval of 3–5 min. All patients were delivered oxygen by face masker and placed in the supine position with a right hip wedge after puncture procedure until the end of the surgical procedure. All BP recordings in this study were performed with the patient in the supine position.

The main objective of this study was to obtain and compare the prevalence of neuraxial anesthesia-related hypotension during cesarean delivery in COVID-19 parturients and control parturients (without COVID-19). Hypotension was defined as the systolic blood pressure (SBP) below 100 mmHg, or the mean arterial blood pressure (MAP) below 80% of the baseline value (the mean of repeated measurements before commencing anesthesia) (17, 18). Given the administration of vasopressors and fluid for prophylactic or treatment of hypotension depended heavily on anaesthesiologists, infusion volume over 1,000 ml or vasoconstrictor utilization were also considered as the presence of hypotension. Neuraxial anesthesia-related hypotension was based on a single episode of defined hypotension from the time of local anesthetic injection until 15 min after delivery of the newborn. The secondary objective was the analysis of anesthetic implementation and administration (full stomach, antiemetic utilization, infusion volume, site of puncture, approach of puncture, frequency of puncture, dose of local anesthetic, vasoconstrictor, and oxytocin utilization), intraoperative vital signs (SBP, MAP, heart rate, and SaPO_2_), intraoperative adverse reactions (hypoxia (SaPO_2_ <95%), arrhythmia, nausea, vomiting, dizziness, and shaking), and neonatal Apgar scores at 1 and 5 min after delivery. The clinical characteristics (COVID-19 diagnosis, signs and symptoms, and preoperative laboratory parameters) of COVID-19 parturients also be analyzed.

We derived PSM to balance the predictors for neuraxial anesthesia-related hypotension based on previous studies (8–11, 19–25). Maternal age, body mass index (BMI), baseline heart rate, baseline MAP, baseline SBP, urgency of surgery, anesthetic technique (including EA, SA, and CES), sensory block height, the time interval between neuraxial anesthesia block and start of surgery (block-surgery time), newborns weight and experience of anaesthesiologists were included in the calculation of propensity scores with multivariate logistic regression in our study. Matching was performed without replacement with a 1: 2 matching protocol and a caliper width of 0.03. The quality of the PSM was assessed in two ways. First, we visually assessed propensity score histograms for two groups before and after PSM. Second, absolute standardized difference (ASD) <10% for a given covariate was considered well-balanced.

All statistical analyses were performed using Stata/SE 15.1 (Stata Corp, College Station, TX, USA). Continuous variables are expressed as Mean ± standard deviation (*SD*) or Median (inter-quartile range, IQR). Categorical variables are expressed as Number (proportion). The Mann-Whitney *U*-test, χ^2^ or Fisher's exact test were used on appropriate. A two-tailed *P* < 0.05 was considered as statistical significance.

## Results

**Figure 1:**
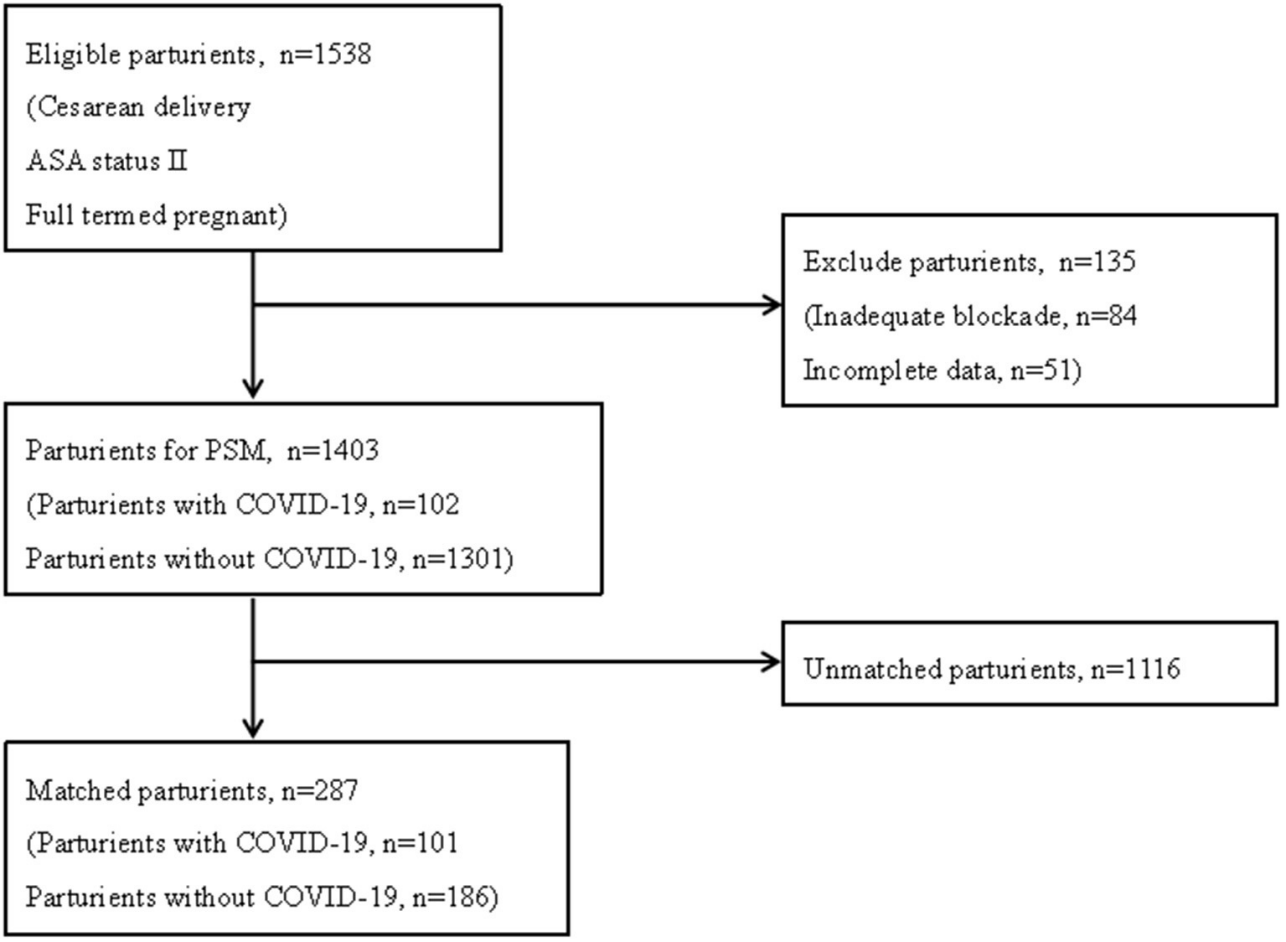
The study flowchart.

The study flowchart was shown in Figure 1. Among 1,538 eligible subjects, we included 1,403 subjects [102 parturients infected with SARS-CoV-2 (COVID-19 group), and 1,301 parturients without (Control group)] for PSM after exclusion of individuals with inadequate blockade (84 cases) or incomplete data (51 cases). After PSM, 287 patients were created, with 101 cases in COVID-19 group and 186 cases in Control group. The characteristics of predictors for neuraxial anesthesia-related hypotension before and after PSM were shown in Table 1. There were statistically significant differences (*P* < 0.05) between COVID-19 and Control groups across several variables before PSM, including urgency of surgery (*P* < 0.01), anesthetic technique (*P* < 0.01), the time interval between EA initiation and surgery start (*P* = 0.02), and anaesthesiologist experience (*P* = 0.02). Parturients with preoperative comorbidities (such as chronic hypertension and hypertensive disorders of pregnancy) which were known to impact maternal blood pressure were not found in the PSM cohort. There were no significant differences between two groups for any of the measured variables after PSM. Meanwhile, ASD for all variables were <10%, indicating that the propensity scores were well-matched (Table 1).

**Table 1:** The characteristics of predictors for neuraxial anesthesia-related hypotension in parturients before and after propensity score matching (PSM).

	**Before PSM**				**After PSM**			
	**COVID-19 (*n* = 102)**	**Control (*n* = 1,301)**	***P*-value**	**ASD (%)**	**COVID-19 (*n* = 101)**	**Control (*n* = 186)**	***P*-value**	**ASD (%)**
Age (years)	28.3 ± 5.9	29.1 ± 5.4	0.16	13.8	28.4 ± 5.9	29.0 ± 5.3	0.39	3.4
BMI (kg/m^2^)	25.7 ± 4.7	25.5 ± 4.4	0.80	−2.5	25.6 ± 4.7	25.3 ± 4.3	0.61	8.9
Baseline heart rate (bpm)	92 ± 14	92 ± 13	0.76	3.1	92 ± 14	93 ± 13	0.45	−0.9
Baseline MAP (mmHg)	83 ± 9	82 ± 9	0.11	−16	83 ± 9	83 ± 10	0.77	6.5
Baseline SBP (mmHg)	117 ± 11	116 ± 11	0.42	−8.5	117 ± 11	11 7± 12	0.84	3.7
Urgency of surgery			<0.01^a^	29.8			0.71	−4.2
Emergency	47 (46.1%)	412 (31.7%)			46 (45.5%)	89 (47.8%)		
Elective	55 (53.9%)	944 (72.6%)			55 (54.5%)	97 (52.2%)		
Anesthetic technique			<0.01^a^	−27.4			0.89	1.9
EA	48 (47.1%)	789 (60.6%)			48 (47.5%)	90 (48.4%)		
CES	54 (52.9%)	512 (39.4%)			53 (52.5%)	96 (51.6%)		
Sensory block height				−17.4				−2.2
EA			0.38				0.37	
T2	0	1 (0.1%)			0	0		
T4	16 (33.3%)	239 (30.3%)			16 (33.3%)	20 (22.2%)		
T6	31 (64.6%)	545 (69.1%)			31 (64.6%)	68 (75.6%)		
T8	1 (2.1%)	3 (0.4%)			1 (2.1%)	2 (2.2%)		
CES			0.05				0.21	
T2	2 (3.7%)	36 (7.0%)			2 (3.8%)	5 (5.2%)		
T4	7 (13.0%)	143 (27.9%)			7 (13.2%)	4 (4.2%)		
T6	45 (83.3%)	331 (64.6%)			44 (83.0%)	72 (75.0%)		
T8	0	2 (0.4%)			0	0		
Block- surgery time (min)				9.9				−3.9
EA	19.7 ± 6.5	17.9 ± 5.1	0.02^a^		19.7 ± 6.5	18.7 ± 5.6	0.35	
CES	6.4 ± 3.1	6.4 ± 2.7	1.00		6.3 ± 3.0	6.5 ± 2.6	0.67	
Newborn weight (kg)	3.5 ± 0.4	3.5 ± 0.5	0.58	6.1	3.5 ± 0.4	3.5 ± 0.5	0.40	−4
Anaesthesiologist experience (years)	5.9 ± 4.0	5.0 ± 3.7	0.02^a^	−22.7	5.8 ± 4.0	5.6 ± 4.0	0.66	−5.1

*Values are Number (proportion) or Mean ± SD. Block- surgery time, the time interval between neuraxial anesthesia block and start of surgery. ASD, absolute standardized difference, the difference in means or proportions divided by the mutual standard deviation and reported as percentages; BMI, body mass index; MAP, mean arterial blood pressure; SBP, systolic blood pressure; EA, epidural anesthesia; CES, combined epidural–spinal anesthesia. The t-test was used to compare COVID-19 group vs. Control group for continuous variables, and χ^2^-test or Fisher's exact test for categorical variables*.

a *Comparing COVID-19 group with Control group, P <0.05*.

As shown in Table 2, the incidence of neuraxial anesthesia-related hypotension was higher in COVID-19 group than that in Control group before and after PSM [57.8 vs. 39.1% (*P* < 0.001); 57.4 vs. 41.9% (*P* = 0.01), respectively]. The incidence risk ratio for neuraxial anesthesia-related hypotension in parturients were 1.48 (95% CI 1.24–1.77) and 1.37 (95% CI 1.08–1.74) before and after PSM, respectively. At the 0.05 significance level, the un-PSM cohort had lower *Cramér's V*-value (0.10 vs. 0.15) than PSM cohort.

**Table 2:** The incidences of neuraxial anesthesia-related hypotension in COVID-19 group vs. Control group before and after propensity score matching (PSM).

**Hypotension**	**Before PSM**	**After PSM**
COVID-19	59 (57.8%)	58 (57.4%)
Control	509 (39.1%)	78 (41.9%)
*P*-value	<0.001^a^	0.01^a^
χ^2^	13.76	6.30
IRR	1.48 (95% CI 1.24–1.77)	1.37 (95% CI 1.08–1.74)
*Cramér's V*	0.10	0.15

*Values are Number (proportion). IRR, incidence risk ratio; CI, confidence interval. The χ^2^-test was used to compare COVID-19 group vs. Control group for categorical variables*.

a *Comparing COVID-19 group with Control group, P <0.05*.

In PSM cohort, COVID-19 parturients had more times of puncture (*P* < 0.001) and higher rate of vasoconstrictor utilization (*P* = 0.02) than Control parturients. However, there were no significant differences between two groups across those variables, including the rate of full stomach, antiemetic and oxytocin utilization, infusion volume and type of fluids, site and approach of puncture, and the dose of local anesthetic (Table 3). COVID-19 group showed significantly higher than Control group in MAP, but not in SBP, heart rate, and SaO_2_%. The incidences of nausea, vomiting, dizziness, and shaking were significantly higher in COVID-19 group than Control group (48.5 vs. 17.2%, *P* < 0.001; 10.9 vs. 4.3%, *P* = 0.03; 18.8 vs. 3.2%, *P* < 0.001; 51.5 vs. 18.3%, *P* < 0.001; respectively). There was no significant difference in the incidence of maternal tachycardia (*P* = 0.46) between two groups (No other arrhythmia was observed). And intraoperative hypoxia wasn't observed in two groups (Table 4). The Apgar score of newborns at 1 min (*P* = 0.04) after birth was significantly lower in COVID-19 group than that in Control group (Table 5), while no significant difference at 5 min (*P* = 0.36).

**Table 3:** Anesthetic implementation and administration in COVID-19 group vs. Control group after propensity score matching (PSM).

	**After PSM**		
	**COVID-19 (*n* = 101)**	**Control (*n* = 186)**	***P*-value**
Full stomach	32 (31.7%)	50 (26.9%)	0.39
Antiemetic utilization			0.45
Metoclopramide	74 (73.3%)	169 (90.7%)	
Metoclopramide and 5-HT3 receptor antagonist	27 (26.7%)	17 (9.3%)	
Infusion volume (ml)			0.08
>1,000	12 (11.9%)	10 (5.4%)	
500–1,000	74 (73.2%)	155 (83.3%)	
<500	15 (14.9%)	21 (11.3%)	
Type of fluids			0.15
Crystalloids	68 (67.3%)	109 (58.6%)	
Crystalloids and Colloids	33 (32.7%)	77 (41.4%)	
Site of puncture			0.94
EA			
L2-3	41 (40.6%)	78 (41.9%)	
L3-4	7 (6.9%)	12 (6.5%)	
CES			
L3-4	38 (37.6%)	73 (39.2%)	
L4-5	15 (14.9)	23 (12.4%)	
Approach of puncture			0.89
EA			
Median	45 (44.5%)	86 (46.2%)	
Lateral	3 (3.0%)	4 (2.2%)	
CES			
Median	53 (52.5%)	96 (51.6%)	
Lateral	0	0	
Frequency of puncture			<0.001^a^
1	75 (74.3%)	173 (93.0%)	
2	26 (25.7%)	11 (5.9%)	
3	0	2 (1.1%)	
CES			
Dose of lidocaine (mg)	0	0	
Dose of ropivacaine (mg)	12.4 ± 1.8	12.1 ± 1.7	0.50
EA			
Dose of lidocaine (mg)	330 ± 38	340 ± 50	0.42
Dose of ropivacaine (mg)	55.0 ± 13.9	59.1 ± 11.6	0.28
Vasoconstrictor utilization			0.02^a^
Methoxamine	17 (16.8%)	22 (11.8%)	
Metaraminol	8 (7.9%)	8 (4.3%)	
Phenylephrine	12 (11.9%)	13 (7.0%)	
Oxytocin utilization			
Peripheral intravenous	101 (100%)	186 (100%)	
Intramyometrial	27 (26.7%)	51 (27.4%)	0.90

*Values are Number (proportion) or Mean ± SD. EA, epidural anesthesia; CES, combined spinal-epidural anesthesia. The t-test was used to compare COVID-19 group vs. Control group for continuous variables, and chi-square test or Fisher's exact test for categorical variables*.

a *Comparing COVID-19 group with Control group, P <0.05*.

**Table 4:** Intraoperative maternal vital signs and adverse reactions in COVID-19 group vs. Control group after propensity score matching (PSM).

	**After PSM**		
	**COVID-19 (*n* = 101)**	**Control (*n* = 186)**	***P*-value**
SBP (mmHg)	113 ± 7	114 ± 8	0.29
MAP (mmHg)	80 ± 8	78 ± 7	0.03^a^
Heart rate (bpm)	84 ± 13	85 ± 11	0.49
SaPO_2_ (%)	99 ± 1	99 ± 1	1.00
Hypoxia (SaPO_2_ <95%)	0	0	
**Arrhythmia**
Tachycardia (heart rate >100 bpm)	18 (17.8%)	40 (21.5%)	0.46
Nausea	49 (48.5%)	32 (17.2)	<0.001^a^
Vomiting	11 (10.9%)	8 (4.3%)	0.03^a^
Dizziness	19 (18.8%)	6 (3.2%)	<0.001^a^
Shaking	52 (51.5%)	34 (18.3%)	<0.001^a^

*Values are Number (proportion). The χ^2^-test or Fisher's exact test was used to compare COVID-19 group vs. Control group for categorical variables*.

a *Comparing COVID-19 group with Control group, P <0.05*.

**Table 5:** Neonatal Apgar scores in COVID-19 group vs. Control group after propensity score matching (PSM).

	**After PSM**		
	**COVID-19 (*n* = 101)**	**Control (*n* = 186)**	***P*-value**
1 min			0.04^a^
10	80 (79.2%)	165 (88.7%)	
9	16 (15.8%)	20 (10.8%)	
8	4 (4.0%)	1 (0.5%)	
7	1 (1%)	0	
5 min			0.36
10	99 (98.0%)	185 (99.5%)	
9	1 (1.0%)	1 (0.5%)	
8	1 (1.0%)	0	

*Values are Number (proportion). The Fisher's exact test was used to compare COVID-19 group vs. Control group for categorical variables*.

a *Comparing COVID-19 group with Control group, P <0.05*.

Among COVID-19 parturients, there were no significant differences in the signs, symptoms, and preoperative laboratory parameters between parturients with neuraxial anesthesia-related hypotension and without (all *P* > 0.05, Supplementary Table 1). Body temperature over 37.4°C (38.6%) and cough (32.7%) were the main signs and symptoms, respectively. The other signs and symptoms included fatigue (9.9%), chest distress (14.6%), dyspnea (8.9%), and diarrhea (6.9%). Significant difference of neonatal Apgar scores at 1 min (*P* = 0.046), but not 5 min (*P* = 0.35), was observed in COVID-19 parturients with neuraxial anesthesia-related hypotension vs. without (Table 6).

**Table 6:** Neonatal Apgar scores from COVID-19 parturients with or without hypotension.

	**COVID-19 parturients**		
	**With hypotension (*n* = 58)**	**Without hypotension (*n* = 43)**	***P*-value**
1 min			0.046^a^
10	41 (70.7%)	39 (90.6%)	
9	14 (24.1%)	2 (4.7%)	
8	2 (3.5%)	2 (4.7%)	
7	1 (1.7%)	0	
5 min			0.35
10	57 (98.3%)	42 (97.7%)	
9	0	1 (2.3%)	
8	1 (1.7%)	0	

*Values are number (proportion). The Fisher's exact test was used to compare COVID-19 group vs. Control group for categorical variables*.

a *Comparing COVID-19 group with Control group, P <0.05*.

## Discussion

In this study, we conducted a retrospective analysis of the incidence of neuraxial anesthesia-related hypotension in parturients with or without COVID-19 undergoing cesarean delivery. Our data suggested that parturients with COVID-19 might experience neuraxial anesthesia-related hypotension more frequently than without.

Previous studies showed that the incidences of hypotension in parturients who underwent a cesarean section were 32–52% after EA, 43–64% after SA, and 32–70% after CES (8–11, 20–22, 26). In present study, the incidences of hypotension after neuraxial anesthesia were ~58 and 40% in parturients with and without COVID-19, respectively. These different results might be attributed to several reasons, such as definition of hypotension, methods and interval time of measurement, and experience of anaesthesiologist (10, 11, 23, 24). Additionally, the short-lived hypotension might also be missed or rapidly recovered by vasoconstrictor pre-treatment. Given the underestimation of risk of hypotension in the retrospective study design, we adopted a loosen hypotension definition in present study.

A recent study found that baby weight, baseline SBP, sensory block height, the time interval between spinal induction and skin incision, and experience of anaesthesiologists were associated with SA-related hypotension in obstetric surgery (11). Maternal age, BMI, weight gain, gravidity, history of hypotension, baseline heart rate, fluid preloading, and anesthetic adjuvant were also demonstrated to be predictive factors for SA-related hypotension in cesarean delivery (10). Chronic alcohol consumption, history of hypertension, ASA physical status, urgency of surgery, surgical department, amount of local anesthetic, and preload liquid were found to have a critical predictive value for the incidence of hypotension in SA and CES for non-obstetric surgery (23, 24). According to evidences above, maternal age, BMI, baseline heart rate, MAP, SBP, urgency of surgery, anesthetic technique, sensory block height, block- surgery time, newborn weight, and anaesthesiologist experience were employed to calculate the propensity scores in present study. However, significant differences were only found in urgency of surgery, anesthetic technique, block- surgery time of EA, and anaesthesiologist experience between COVID-19 group and Control group in un-PSM cohort of present study. The effects of COVID-19 epidemic on urgency of surgery and the choice of anesthetic technique should be taken into account during anesthesia and investigated in future studies.

There was a higher incidence of hypotension in obese parturients during neuraxial anesthesia due to vascular compression by hypertrophic uterus (27). A left-tilt position has been commonly used in parturients, particularly in obese parturients. Nevertheless, a recent study declared that the hemodynamic parameters derived from a non-invasive cardiac output monitoring system were not statistically different between the left-tilt and supine position (28). Pre-administration of vasoconstrictor and volume most likely concealed the appearance of hypotension (18, 29, 30).

Consistent with previous studies, our data also revealed anaesthesiologist experience in association with the risk of neuraxial anesthesia-related hypotension (11). An experienced anaesthesiologist can protect parturients with low baseline BP or high level of sensory blockade from high risk of hypotension. Intriguingly, there was a significantly higher incidence of hypotension in COVID-19 group than that in Control group before PSM, although those anaesthesiologists had more experience. In the PSM cohort, experience of anaesthesiologists was comparable in two groups, but COVID-19 parturients still had a higher incidence of neuraxial anesthesia-related hypotension and vasoconstrictor utilization. Those results supported COVID-19 parturients were at an increased risk for neuraxial anesthesia-related hypotension. Additionally, COVID-19 parturients suffered more times of puncture in present study. Part of this difference seems due to the personal protective equipment of anaesthesiologists.

ACE2 dysfunction induced by SARS-CoV-2 infection was referred to virus toxicity, hypoxia status, inflammation, and sympathetic hyperactivity (31), which might increase the susceptibility of neuraxial anesthesia-related hypotension resulted from sympathetic inhibition in COVID-19 parturients. Although clinical practices have demonstrated that neuraxial anesthesia is a safe technique for obstetric anesthesia in COVID-19 parturients (5, 6, 32, 33), higher incidences of hypotension and discomforts, and lower Apgar scores at 1 min were observed in COVID-19 parturients and their babies in present study. More evidences derived from a larger sample size and randomized controlled trails are also needed to determine the effects and mechanisms of COVID-19 on hemodynamics of parturients undergoing neuraxial anesthesia for cesarean delivery.

Clinical practice, such as anesthetic technique, local anesthetic concentration, vasopressor, and volume administration, were not sufficiently standardized in this multicenter retrospective study in order to draw conclusive conclusions. And, a *post-hoc* estimated power value 0.71 and effect size (*Cramér's V-*value) 0.15 after PSM would be another limitation. However, an increased risk of neuraxial anesthesia-related hypotension in COVID-19 parturients undergoing cesarean delivery should be stressed.

## Data Availability Statement

The original contributions presented in the study are included in the article/Supplementary Material, further inquiries can be directed to the corresponding author/s.

## Ethics Statement

The studies involving human participants were reviewed and approved by Institutional Review Board at Renmin Hospital of Wuhan University, Wuhan, China (Chairperson Prof. Hong Chen). Written informed consent for participation was not required for this study in accordance with the national legislation and the institutional requirements.

## Author Contributions

QM and XC designed the study. YZ, RC, CC, YG, and QZ collected the data. CC and YG analyzed and interpreted the work. YZ and RC drafted the manuscript. ZX and MW revised it critically for important intellectual content. All authors contributed to the manuscript and approved the final version.

## Conflict of Interest

The authors declare that the research was conducted in the absence of any commercial or financial relationships that could be construed as a potential conflict of interest.

## Publisher's Note

All claims expressed in this article are solely those of the authors and do not necessarily represent those of their affiliated organizations, or those of the publisher, the editors and the reviewers. Any product that may be evaluated in this article, or claim that may be made by its manufacturer, is not guaranteed or endorsed by the publisher.

## Supplementary Material

Supplementary Figure 1 Summaries of the balance of covariates before and after propensity score matching. Absolute standardized difference <10% for a given covariate was considered well balanced. Block- surgery time, the time interval between neuraxial anesthesia block and start of surgery; BMI, body mass index; SBP, systolic blood pressure; MAP, mean arterial blood pressure.

Supplementary Table 1 Clinical characteristics of COVID-19 parturients with or without hypotension. Values are Number (proportion) or Median (IQR).
