# Supplementary figures and images for: The Risk of Neuraxial Anesthesia-Related Hypotension in COVID-19 Parturients Undergoing Cesarean Delivery: A Multicenter, Retrospective, Propensity Score Matched Cohort Study
Source: Front Med (Lausanne). 2021 Aug 19;8:713733. doi: 10.3389/fmed.2021.713733 (PMC8416900; doi:10.3389/fmed.2021.713733)

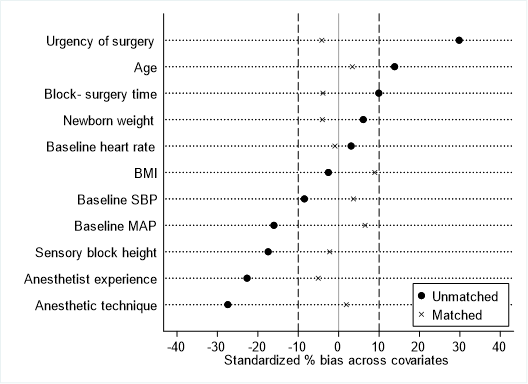

Supplement: Supplementary Figure 1 — Summaries of the balance of covariates before and after propensity score matching. Absolute standardized difference <10% for a given covariate was considered well balanced. Block- surgery time, the time interval between neuraxial anesthesia block and start of surgery; BMI, body mass index; SBP, systolic blood pressure; MAP, mean arterial blood pressure. [file Image_1.TIF]
