# Supplementary material for: The Risk of Neuraxial Anesthesia-Related Hypotension in COVID-19 Parturients Undergoing Cesarean Delivery: A Multicenter, Retrospective, Propensity Score Matched Cohort Study
Source: Front Med (Lausanne). 2021 Aug 19;8:713733. doi: 10.3389/fmed.2021.713733 (PMC8416900; doi:10.3389/fmed.2021.713733)
Supplement: Supplementary Table 1 — Clinical characteristics of COVID-19 parturients with or without hypotension. Values are Number (proportion) or Median (IQR). [file Table_1.DOCX]

**Supplemental table 1.** Clinical characteristics of COVID-19 parturients with or without hypotension. Values are Number (proportion) or Median (IQR).

|  | COVID-19 parturients | | | |
| --- | --- | --- | --- | --- |
|  | All cohort  (101) | With hypotension  (n=58) | Without hypotension (n=43) | *P*-value |
| COVID-19 diagnose |  |  |  |  |
| SARS-CoV-2 nucleic acid test positive | 101 | 58 (100%) | 43 (100%) |  |
| CT evidence of pneumonia | 101 | 58 (100%) | 43 (100%) |  |
| Signs and symptoms |  |  |  |  |
| Fever | 39 (38.6%) | 22 (37.9%) | 17 (39.5%) | 0.87 |
| Cough | 33 (32.7%) | 15 (25.9%) | 18 (41.9%) | 0.09 |
| Fatigue | 10 (9.9%) | 4 (6.9%) | 6 (14.0%) | 0.24 |
| Chest distress | 15 (14.6%) | 7 (12.1%) | 8 (18.6%) | 0.36 |
| Dyspnoea | 9 (8.9%) | 3 (3.0%) | 6 (3.2%) | 0.13 |
| Diarrhea | 7 (6.9%) | 4 (6.9%) | 3 (7.0%) | 0.99 |
| Preoperative laboratory parameters |  |  |  |  |
| Hemoglobin (g/L) | 104 (95 to 128) | 107 (94 to 128) | 103 (96 to 125) | 0.98 |
| White blood cell count (× 10^9^/L) | 8.0 (5.0 to 9.0) | 8.3 (4.7 to 9.0) | 7.8 (5.4 to 9.1) | 0.90 |
| Lymphocyte count (× 10^9^/L) | 2.4 (1.4 to 3.9) | 2.6 (1.0 to 4.1) | 2.3 (1.6 to 3.8) | 0.82 |
| CRP (mg/L) | 2.8 (1.5 to 5.4) | 3.1(1.6 to 6.0) | 2.7 (1.5 to 5.2) | 0.82 |
| ALT (U/L) | 35 (20 to 51) | 35 (21 to 47) | 35 (18 to 55) | 0.79 |
| AST (U/L) | 29 (16 to 48) | 30 (18 to 42) | 26 (14 to 49) | 0.53 |
| BUN (mmol/L) | 4.7 (3.4 to 9.4) | 4.6 (3.4 to 9.0) | 5.2 (3.1 to 10.1) | 0.46 |
| SCr (μmol/L) | 61 (36 to 89) | 57 (30 to 87) | 62 (45 to 93) | 0.05 |

Notes: Fever, body temperature ≥ 37.4℃; Chest distress, a stuffy feeling in the chest. The *χ^2^* test or Fisher’s exact test was used to compare with hypotension group *vs* without hypotension group for categorical variables, Mann-Whitney *U* test for continuous variables.
